# Conservation laws, solitary wave solutions, and lie analysis for the nonlinear chains of atoms

**DOI:** 10.1038/s41598-023-38658-w

**Published:** 2023-07-17

**Authors:** Muhammad Junaid-U-Rehman, Grzegorz Kudra, Jan Awrejcewicz

**Affiliations:** Department of Automation, Biomechanics, and Mechatronics, Lodz University of Technology, 1/15 Stefanowski St. (Building A22), 90-924 Lodz, Poland

**Keywords:** Mechanical engineering, Mathematics and computing, Applied mathematics, Computational science, Software

## Abstract

Nonlinear chains of atoms (NCA) are complex systems with rich dynamics, that influence various scientific disciplines. The lie symmetry approach is considered to analyze the NCA. The Lie symmetry method is a powerful mathematical tool for analyzing and solving differential equations with symmetries, facilitating the reduction of complexity and obtaining solutions. After getting the entire vector field by using the Lie scheme, we find the optimal system of symmetries. We have converted assumed PDE into nonlinear ODE by using the optimal system. The new auxiliary scheme is used to find the Travelling wave solutions, while graphical behaviour visually represents relationships and patterns in data or mathematical models. The multiplier method enables the identification of conservation laws, and fundamental principles in physics that assert certain quantities remain constant over time.

## Introduction

The Lie symmetry analysis approach^1–12^ has many applications in different fields, including physics, engineering, and mathematical modelling. It can be used to study a wide range of nonlinear PDEs, including those that are difficult to solve using other methods. Additionally, this approach provides a powerful tool for developing new theories and models that improve our understanding of complex physical systems. Overall, the Lie symmetry analysis approach is a valuable tool for studying nonlinear PDEs and has many significant applications in different branches of science and engineering.

The Lie symmetry analysis approach^13–23^ is a powerful method used in the study of nonlinear PDEs. It is based on the concept of Lie groups and Lie algebras, which are mathematical structures that describe the symmetries of a system. The Lie symmetry analysis approach involves transforming a given PDE into an equivalent system of ODEs using a Lie group transformation. This transformation is constructed from a set of symmetry generators that preserve the form of the original PDE. Once the PDE is transformed into an equivalent system of ODEs, it is possible to use various analytical and numerical methods to solve the system and obtain the solution to the original PDE. Additionally, the Lie symmetry analysis approach can be used to identify the conservation laws that govern the physical behaviour of the system under study. These conservation laws provide important insights into the underlying physical mechanisms responsible for the observed behaviour of the system.

Nonlinear PDEs^24–27^ play a critical role in mechanical engineering by modelling complex phenomena such as stress and deformation in materials, fluid flow, and heat transfer. Unlike linear PDEs, which can be solved analytically in many cases, nonlinear PDEs require numerical or approximate methods to solve due to their complex nature. The use of nonlinear PDEs is essential in the design and optimization of mechanical systems such as turbines, engines, and aircraft. They also provide a framework for predicting the behaviour of materials under different conditions, such as high temperatures, high pressure, and rapid deformation. By incorporating nonlinear PDEs into mechanical engineering models, engineers can improve the accuracy of their designs and ensure that their systems are safe, reliable, and efficient.

Nonlinear chains of atoms^28–34^ have a wide range of applications in mechanical engineering, particularly in the study of materials science and solid mechanics. These models provide valuable insights into the behaviour of materials at the atomic level, enabling the design of high-performance materials for various applications. Applications of nonlinear chains of atoms include the study of thermal conductivity in materials, investigating the deformation and fracture mechanisms of materials under various loading conditions, and studying the dynamics of crystals, such as the propagation of waves and the formation of defects. Nonlinear chains of atoms models are crucial in understanding the behaviour of materials under extreme conditions and can inform the design of materials for high-performance applications, leading to the development of new materials with enhanced mechanical and thermal properties.

The analysis of nonlinear chains of atoms using Lie symmetry analysis and conservation laws is motivated by the desire to uncover the fundamental principles that govern the behaviour of these complex systems and gain a deeper understanding of their dynamics and properties. While there has been extensive research on nonlinear chains of atoms, there exist research gaps that can be addressed through the application of Lie symmetry analysis and conservation laws. These gaps include a need for comprehensive studies on the symmetries and conservation laws specific to nonlinear atomic chains, exploration of multiscale behaviour, and bridging the gap between theoretical analysis and practical applications. By addressing these research gaps, researchers can contribute to a more comprehensive understanding of nonlinear chains of atoms and pave the way for advancements in fields such as materials design, nanotechnology, and engineering applications.

The new auxiliary method^35, 36^ is a recently proposed method for solving challenging nonlinear PDEs. This method involves introducing an auxiliary variable and constructing a system of coupled equations involving both the original variables and the auxiliary variable. The resulting system of equations can be solved using numerical methods to obtain the solution to the original PDE. The new auxiliary method can handle highly nonlinear PDEs that are difficult to solve using other numerical methods, such as the finite difference approach or the finite element scheme. Additionally, this method can be used to obtain exact solutions to certain types of nonlinear PDEs, reducing the computational cost required to solve some types of nonlinear PDEs. Overall, the new auxiliary method is a promising tool for solving challenging nonlinear PDEs in various fields, including physics, engineering, and mathematical modelling.

Conservation laws of nonlinear PDEs^37–40^ are essential concepts that relate to the principle of conservation of physical quantities like mass, energy, and momentum. These laws are expressed in terms of PDEs and have crucial importance in various fields, including engineering, physics, and mathematical modelling. They provide a mathematical framework to predict the behaviour of complex physical systems accurately and develop new theories and models to improve our understanding of the underlying physical mechanisms. Furthermore, conservation laws play a vital role in the design and analysis of physical systems and the development of numerical methods for solving challenging nonlinear PDEs, making them fundamental concepts in the study of nonlinear PDEs.

The layout of this research is presented as. Formation of supposed model is described in section 2. NAM and multiplier method are explained in section 3. technique and multiplier scheme are presented in section 2. Lie group analysis method is applied on supposed model and entire vector field is described in section 4. Optimal system, similarity reduction, wave solutions, and graphics are represented in section 5. Discussion of graphs and conservation laws of assumed model are described in section 6 and 7 respectively. The conclusions are stated in Sect. 8.

## Formation of model

The Hamiltonian of the system is Foroutan et al.^41^:

1 $$\begin{aligned} H=\sum _n{\Bigg \{\sum _{l\ne n} {\mathcal {V}}(|{\mathcal {U}}_n-{\mathcal {U}}_l|)+\frac{1}{2}m{\mathcal {U}}_n^{.2}\Bigg \}}, \end{aligned}$$

where *m* is the mass of the atom, ${\mathcal {V}}(|{\mathcal {U}}_n-{\mathcal {U}}_l|)$ stands for nonlinear potential and dot indicates for derivative w.r.t time. We consider $l=1\pm n$ and the subsequent potential:

2 $$\begin{aligned} {\mathcal {V}}(h_{nl})=\frac{1}{4}\beta _{i}h^4_{nl}+\frac{1}{3}\alpha _i h^3_{nl}+ \frac{1}{2}\gamma _i h^2_{nl}, \end{aligned}$$

where $h_{nl}$ is relative displacement among $l-th$ atom and $n-th$. The index *i* shows the distinct interactions via the particles. We omit our focus on the first and second neighbours. From Eqs. (1) and (2) through Hamiltonian equations which are

$$\begin{aligned} \frac{\partial H}{\partial {\mathcal {U}}_n}=-P_n^{.},~~~~~~\frac{\partial H}{\partial P^{.}_n}=\frac{\partial {\mathcal {U}}_n}{\partial \tau }={\mathcal {U}}^{.}_n, \end{aligned}$$

which gives us the equation of motion;

3 $$\begin{aligned} \begin{aligned} \frac{d^2 {\mathcal {U}}_n}{d \tau ^2}&=\gamma _1\big ({\mathcal {U}}_{n+1}-2{\mathcal {U}}_n+{\mathcal {U}}_{n-1}\big )\\&\quad +\gamma _2\big ({\mathcal {U}}_{n+2}-2{\mathcal {U}}_n+{\mathcal {U}}_{n-2}\big )\\&\quad +\alpha _1\bigg \{\big ({\mathcal {U}}_{n+1}-{\mathcal {U}}_n\big )^2-\big ({\mathcal {U}}_{n}-{\mathcal {U}}_{n-1}\big )^2\bigg \}\\&\quad +\alpha _2\bigg \{\big ({\mathcal {U}}_{n+2}-{\mathcal {U}}_n\big )^2-\big ({\mathcal {U}}_{n}-{\mathcal {U}}_{n-2}\big )^2\bigg \}\\&\quad +\beta _1\bigg \{\big ({\mathcal {U}}_{n+1}-{\mathcal {U}}_n\big )^2-\big ({\mathcal {U}}_{n}-{\mathcal {U}}_{n-1}\big )^2\bigg \}\\&\quad +\beta _2\bigg \{\big ({\mathcal {U}}_{n+2}-{\mathcal {U}}_n\big )^2-\big ({\mathcal {U}}_{n}-{\mathcal {U}}_{n-2}\big )^2\bigg \}. \end{aligned} \end{aligned}$$

In Hamiltonian’s equations, $P^{.}_n$ stands for generalized momentum. Assuming that the $\delta$(inter-atom spacing) is small enough so that the continuum limit is reached, we substitute $\delta _n\rightarrow {\chi }$. Then

4 $$\begin{aligned} {\mathcal {U}}_{n\pm 1}={\mathcal {U}}\pm \delta {\mathcal {U}}_\chi +\frac{1}{2}\delta ^2{\mathcal {U}}_{\chi \chi }\pm \frac{1}{6}\delta ^3 {\mathcal {U}}_{\chi \chi \chi }+\frac{1}{24}\delta ^4{\mathcal {U}}_{\chi \chi \chi \chi }+ \cdots , \end{aligned}$$

and

5 $$\begin{aligned} {\mathcal {U}}_{n\pm 2}={\mathcal {U}}\pm 2\delta {\mathcal {U}}_\chi +\frac{4}{2}\delta ^2 {\mathcal {U}}_{\chi \chi }\pm \frac{8}{6}\delta ^3 {\mathcal {U}}_{\chi \chi \chi }+\frac{16}{24}\delta ^4 {\mathcal {U}}_{\chi \chi \chi \chi }+ \cdots , \end{aligned}$$

hence, Eq. (3) can be supposed as Foroutan et al.^42^

6 $$\begin{aligned} \frac{\partial ^2 {\mathcal {U}}}{\partial \tau ^2}=\delta ^2_{o}\frac{\partial ^2 {\mathcal {U}}}{\partial \chi ^2}+p_{o} \frac{\partial {\mathcal {U}}}{\partial \chi } \frac{\partial ^2 {\mathcal {U}}}{\partial \chi ^2}+q_{o} \bigg (\frac{\partial {\mathcal {U}}}{\partial \chi }\bigg )^2 \frac{\partial ^2 {\mathcal {U}}}{\partial \chi ^2}+r\frac{\partial ^4 {\mathcal {U}}}{\partial \chi ^4}, \end{aligned}$$

with the subsequent constants;

7 $$\begin{aligned} \begin{aligned} \delta ^2_{o}=\frac{\delta ^2}{m}\big (\gamma _1+4\gamma _2\big ),~p_{o} =\frac{2\delta ^3}{m}\big (\gamma _1+8\alpha _2\big ),~q_{o}=\frac{3\delta ^4}{m}\big (\beta _1+16\beta _2\big ), r=\frac{\delta ^4}{12m}\big (\gamma _1+16\gamma _2\big ). \end{aligned} \end{aligned}$$

here in this paper, we will find out wave solutions and conservation laws for nonlinear Eq. (6) with the use of an appropriate transformation method.

## Preliminaries

### New auxiliary approach

Assuming the general form of partial PDE is of the form:

8 $$\begin{aligned} \mathcal {F}({\mathcal {U}},{\mathcal {U}}_\tau ,{\mathcal {U}}_{\chi }, {\mathcal {U}}_{\chi \chi },\dots )=0, \end{aligned}$$

where $\tau$ is the time part and $\chi$ is the spatial part and ${\mathcal {U}}={\mathcal {U}}(\chi ,\tau )$ is the dependent variable. We will follow the following steps.

- *Step 1* Suppose the new similarity variables or transformation is of the form
  9 $$\begin{aligned} {\mathcal {U}}(\chi ,\tau )={\mathcal {H}}(\varrho ),~~~~\text {where}~~~~ \varrho =k(\chi +c\tau ), \end{aligned}$$
  where *k* and *c* both are actual parameters for Eq. (8). Putting the Eq. (9) into Eq. (8) and we get the new ODE below.
  10 $$\begin{aligned} \mathcal {P}(\mathcal {H},{\mathcal {H}}^\prime ,{\mathcal {H}}^{\prime \prime },\dots )=0. \end{aligned}$$
- *Step 2* Assume the general solution for Eq. (10) is of the form
  11 $$\begin{aligned} {\mathcal {H}}(\varrho )=\sum _{i=0}^{N}C_i{\mathfrak {F}}^{iq(\varrho )}, \end{aligned}$$
  in the above solution, the $C_{i}$’s are constants and we will fine later and the 1*st* ODE satisfied $q(\varrho )$.
  12 $$\begin{aligned} q^\prime (\varrho )=\frac{1}{\ln ({\mathfrak {F}})}\{{\mathfrak {B}_{2}} {\mathfrak {F}}^{-q(\varrho )}+{\mathfrak {B}_{1}}+{\mathfrak {B}_{3}} {\mathfrak {F}}^{q(\varrho )}\},~~{\mathfrak {F}}>0,~~{\mathfrak {F}}\ne 1. \end{aligned}$$
- *Step 3* In this step, we will use the balancing scheme to execute the value of *N*. For this, we have to compare the highest-order linear and nonlinear terms to find the value of *N*.
- *Step 4* Getting the coefficients of the powers of ${{\mathfrak {F}}^{q(\varrho )}}$ ($i=0,1,2,3, \dots$) by Eqs. (8), (11), and (12). Then collecting the terms of the same power and put them equal to zero which gives us a system of algebraic equations. After solving this system of equations by *Maple*.
- *Step 5* Finally we will get the different family of solutions for Eq. (12) of the form:

- *Case 1* When ${\mathfrak {B}_{1}}^2-{\mathfrak {B}_{2}}{\mathfrak {B}_{3}}<0$ and ${\mathfrak {B}_{3}}\ne 0$
  13 $$\begin{aligned} {\mathfrak {F}}^{q(\varrho )}=\frac{-{\mathfrak {B}_{1}}}{{\mathfrak {B}_{3}}} +\frac{\sqrt{-({\mathfrak {B}_{1}}^2-{\mathfrak {B}_{2}}{\mathfrak {B}_{3}})}}{{\mathfrak {B}_{3}}}\tan \left( \frac{\sqrt{-({\mathfrak {B}_{1}} ^2-{\mathfrak {B}_{2}}{\mathfrak {B}_{3}})}}{2}\varrho \right) , \end{aligned}$$
  14 $$\begin{aligned} {\mathfrak {F}}^{q(\varrho )}=\frac{-{\mathfrak {B}_{1}}}{{\mathfrak {B}_{3}}} +\frac{\sqrt{-({\mathfrak {B}_{1}}^2 -{\mathfrak {B}_{2}}{\mathfrak {B}_{3}})}}{{\mathfrak {B}_{3}}}\cot \left( \frac{\sqrt{-({\mathfrak {B}_{1}} ^2-{\mathfrak {B}_{2}}{\mathfrak {B}_{3}})}}{2}\varrho \right) . \end{aligned}$$
- *Case 2* When ${\mathfrak {B}_{1}}^2-{\mathfrak {B}_{2}}{\mathfrak {B}_{3}}>0$ and ${\mathfrak {B}_{3}}\ne 0$
  15 $$\begin{aligned} {\mathfrak {F}}^{q(\varrho )}=\frac{-{\mathfrak {B}_{1}}}{{\mathfrak {B}_{3}}} +\frac{\sqrt{ \left( {\mathfrak {B}_{1}}^2-{\mathfrak {B}_{2}}{\mathfrak {B}_{3}} \right) }}{{\mathfrak {B}_{3}}} \tanh \left( \frac{\sqrt{({\mathfrak {B}_{1}} ^2-{\mathfrak {B}_{2}}{\mathfrak {B}_{3}})}}{2}\varrho \right) , \end{aligned}$$
  16 $$\begin{aligned} {\mathfrak {F}}^{q(\varrho )}=\frac{-{\mathfrak {B}_{1}}}{{\mathfrak {B}_{3}}} -\frac{\sqrt{ \left( {\mathfrak {B}_{1}}^2-{\mathfrak {B}_{2}}{\mathfrak {B}_{3}} \right) }}{{\mathfrak {B}_{3}}}\coth \left( \frac{\sqrt{({\mathfrak {B}_{1}} ^2-{\mathfrak {B}_{2}}{\mathfrak {B}_{3}})}}{2}\varrho \right) . \end{aligned}$$
- *Case 3* When ${\mathfrak {B}_{1}}^2+{\mathfrak {B}_{2}}{\mathfrak {B}_{3}}>0$ and ${\mathfrak {B}_{3}}\ne 0$ and ${\mathfrak {B}_{3}}\ne -{\mathfrak {B}_{2}}$
  17 $$\begin{aligned} {\mathfrak {F}}^{q(\varrho )}=\frac{{\mathfrak {B}_{1}}}{{\mathfrak {B}_{3}}} +\frac{\sqrt{\left( {\mathfrak {B}_{1}}^2+{\mathfrak {B}_{2}}^2 \right) }}{{\mathfrak {B}_{3}}} \tanh \left( \frac{\sqrt{ \left( {\mathfrak {B}_{1}}^2+{\mathfrak {B}_{2}}^2 \right) }}{2}\varrho \right) , \end{aligned}$$
  18 $$\begin{aligned} {\mathfrak {F}}^{q(\varrho )}=\frac{{\mathfrak {B}_{1}}}{{\mathfrak {B}_{3}}} +\frac{\sqrt{ \left( {\mathfrak {B}_{1}}^2+{\mathfrak {B}_{2}}^2 \right) }}{{\mathfrak {B}_{3}}}\coth \left( \frac{\sqrt{ \left( {\mathfrak {B}_{1}}^2+{\mathfrak {B}_{2}}^2 \right) }}{2}\varrho \right) . \end{aligned}$$
- *Case 4* When ${\mathfrak {B}_{1}}^2+{\mathfrak {B}_{2}}{\mathfrak {B}_{3}}<0$, ${\mathfrak {B}_{3}}\ne 0$ and ${\mathfrak {B}_{3}}\ne -{\mathfrak {B}_{2}}$
  19 $$\begin{aligned} {\mathfrak {F}}^{q(\varrho )}=\frac{{\mathfrak {B}_{1}}}{{\mathfrak {B}_{3}}} +\frac{\sqrt{- \left( {\mathfrak {B}_{1}}^2+{\mathfrak {B}_{2}}^2 \right) }}{{\mathfrak {B}_{3}}}\tan \left( \frac{\sqrt{- \left( {\mathfrak {B}_{1}}^2+{\mathfrak {B}_{2}}^2 \right) }}{2}\varrho \right) , \end{aligned}$$
  20 $$\begin{aligned} {\mathfrak {F}}^{q(\varrho )}=\frac{{\mathfrak {B}_{1}}}{{\mathfrak {B}_{3}}} +\frac{\sqrt{- \left( {\mathfrak {B}_{1}}^2+{\mathfrak {B}_{2}}^2 \right) }}{{\mathfrak {B}_{3}}}\cot \left( \frac{\sqrt{-({\mathfrak {B}_{1}}^2+{\mathfrak {B}_{2}}^2)}}{2}\varrho \right) . \end{aligned}$$
- *Case 5* When ${\mathfrak {B}_{1}}^2-{\mathfrak {B}_{2}}^2<0$ and ${\mathfrak {B}_{3}}\ne -{\mathfrak {B}_{2}}$
  21 $$\begin{aligned} {\mathfrak {F}}^{q(\varrho )}=\frac{-{\mathfrak {B}_{1}}}{{\mathfrak {B}_{3}}} +\frac{\sqrt{- \left( {\mathfrak {B}_{1}}^2-{\mathfrak {B}_{2}}^2 \right) }}{{\mathfrak {B}_{3}}}\tan \left( \frac{\sqrt{- \left( {\mathfrak {B}_{1}}^2-{\mathfrak {B}_{2}}^2 \right) }}{2}\varrho \right) , \end{aligned}$$
  22 $$\begin{aligned} {\mathfrak {F}}^{q(\varrho )}=\frac{-{\mathfrak {B}_{1}}}{{\mathfrak {B}_{3}}} +\frac{\sqrt{- \left( {\mathfrak {B}_{1}}^2-{\mathfrak {B}_{2}}^2 \right) }}{{\mathfrak {B}_{3}}}\cot \left( \frac{\sqrt{- \left( {\mathfrak {B}_{1}}^2-{\mathfrak {B}_{2}}^2 \right) }}{2}\varrho \right) . \end{aligned}$$
- *Case 6* When ${\mathfrak {B}_{1}}^2-{\mathfrak {B}_{2}}^2>0$ and ${\mathfrak {B}_{3}}\ne -{\mathfrak {B}_{2}}$
  23 $$\begin{aligned} {\mathfrak {F}}^{q(\varrho )}=\frac{-{\mathfrak {B}_{1}}}{{\mathfrak {B}_{3}}} +\frac{\sqrt{ \left( {\mathfrak {B}_{1}}^2-{\mathfrak {B}_{2}}^2 \right) }}{{\mathfrak {B}_{3}}}\tanh \left( \frac{\sqrt{ \left( {\mathfrak {B}_{1}}^2-{\mathfrak {B}_{2}}^2 \right) }}{2}\varrho \right) , \end{aligned}$$
  24 $$\begin{aligned} {\mathfrak {F}}^{q(\varrho )}=\frac{-{\mathfrak {B}_{1}}}{{\mathfrak {B}_{3}}} +\frac{\sqrt{ \left( {\mathfrak {B}_{1}}^2-{\mathfrak {B}_{2}}^2 \right) }}{{\mathfrak {B}_{3}}}\coth \left( \frac{\sqrt{ \left( {\mathfrak {B}_{1}}^2-{\mathfrak {B}_{2}}^2 \right) }}{2}\varrho \right) . \end{aligned}$$
- *Case 7* When ${\mathfrak {B}_{2}}{\mathfrak {B}_{3}}>0$, ${\mathfrak {B}_{3}}\ne 0$ and ${\mathfrak {B}_{1}}=0$
  25 $$\begin{aligned} {\mathfrak {F}}^{q(\varrho )}=\sqrt{\frac{-{\mathfrak {B}_{2}}}{{\mathfrak {B}_{3}}}}\tanh \bigg (\frac{\sqrt{-{\mathfrak {B}_{2}}{\mathfrak {B}_{3}}}}{2}\varrho \bigg ), \end{aligned}$$
  26 $$\begin{aligned} {\mathfrak {F}}^{q(\varrho )}=\sqrt{\frac{-{\mathfrak {B}_{2}}}{{\mathfrak {B}_{3}}}}\coth \left( \frac{\sqrt{-{\mathfrak {B}_{2}}{\mathfrak {B}_{3}}}}{2}\varrho \right) . \end{aligned}$$
- *Case 8* When ${\mathfrak {B}_{1}}=0$ and ${\mathfrak {B}_{2}}=-{\mathfrak {B}_{3}}$
  27 $$\begin{aligned} {\mathfrak {F}}^{q(\varrho )}=\frac{- \left( 1+e^{2{\mathfrak {B}_{2}}\varrho } \right) \pm \sqrt{2 \left( 1+e^{2{\mathfrak {B}_{2}}\varrho } \right) }}{e^{2{\mathfrak {B}_{2}}\varrho }-1}. \end{aligned}$$
- *Case 9* When $${\mathfrak {B}_{1}}^2={\mathfrak {B}_{2}}{\mathfrak {B}_{3}}$$
  28 $$\begin{aligned} {\mathfrak {F}}^{q(\varrho )}=\frac{-{\mathfrak {B}_{2}} \left( {\mathfrak {B}_{1}}\varrho +2 \right) }{{\mathfrak {B}_{1}}^2\varrho }. \end{aligned}$$
- *Case 10* When ${\mathfrak {B}_{1}}=k$, ${\mathfrak {B}_{2}}=2k$ and ${\mathfrak {B}_{3}}=0$
  29 $$\begin{aligned} {\mathfrak {F}}^{q(\varrho )}=e^{\varrho }-1. \end{aligned}$$
- *Case 11* When ${\mathfrak {B}_{1}}=k$, ${\mathfrak {B}_{3}}=2k$ and ${\mathfrak {B}_{2}}=0$
  30 $$\begin{aligned} {\mathfrak {F}}^{q(\varrho )}=\frac{e^{\varrho }}{1-e^{\varrho }}. \end{aligned}$$
- *Case 12* When $$2{\mathfrak {B}_{1}}={\mathfrak {B}_{2}}+{\mathfrak {B}_{3}}$$
  31 $$\begin{aligned} {\mathfrak {F}}^{q(\varrho )}=\frac{1+{\mathfrak {B}_{2}} e^{\frac{1}{2} \left( {\mathfrak {B}_{2}}-{\mathfrak {B}_{3}} \right) \varrho }}{ 1+{\mathfrak {B}_{3}} e^{\frac{1}{2} \left( {\mathfrak {B}_{2}}-{\mathfrak {B}_{3}} \right) \varrho }}. \end{aligned}$$
- *Case 13* When $$-2{\mathfrak {B}_{1}}={\mathfrak {B}_{2}}+{\mathfrak {B}_{3}}$$
  32 $$\begin{aligned} {\mathfrak {F}}^{q(\varrho )}=\frac{{\mathfrak {B}_{2}}+{\mathfrak {B}_{2}} e^{\frac{1}{2} \left( {\mathfrak {B}_{2}}-{\mathfrak {B}_{3}} \right) \varrho }}{ {\mathfrak {B}_{3}}+{\mathfrak {B}_{3}} e^{\frac{1}{2} \left( {\mathfrak {B}_{2}}-{\mathfrak {B}_{3}} \right) \varrho }}. \end{aligned}$$
- *Case 14* When $${\mathfrak {B}_{2}}=0$$
  33 $$\begin{aligned} {\mathfrak {F}}^{q(\varrho )}=\frac{{\mathfrak {B}_{1}} e^{{\mathfrak {B}_{1}}\varrho }}{ 1+\frac{{\mathfrak {B}_{3}}}{2}e^{{\mathfrak {B}_{1}}\varrho }}. \end{aligned}$$
- *Case 15* When $${\mathfrak {B}_{2}}={\mathfrak {B}_{1}}={\mathfrak {B}_{3}}\ne 0$$
  34 $$\begin{aligned} {\mathfrak {F}}^{q(\varrho )}=\frac{- \left( {\mathfrak {B}_{2}}\varrho +2 \right) }{{\mathfrak {B}_{2}}\varrho }. \end{aligned}$$
- *Case 16* When $${\mathfrak {B}_{2}}={\mathfrak {B}_{3}}$$, $${\mathfrak {B}_{1}}=0$$
  35 $$\begin{aligned} {\mathfrak {F}}^{q(\varrho )}=\tan \left( \frac{{\mathfrak {B}_{2}}\varrho +c}{2} \right) . \end{aligned}$$
- *Case 17* When $${\mathfrak {B}_{3}}=0$$
  36 $$\begin{aligned} {\mathfrak {F}}^{q(\varrho )}=e^{{\mathfrak {B}_{1}}\varrho }-\frac{{\mathfrak {B}_{2}}}{2{\mathfrak {B}_{1}}}. \end{aligned}$$

### Multiplier approach

Supposing the Eq. (8) and applying the following steps below:

1. Defining the total differential as:
  37 $$\begin{aligned} D_{i}=\frac{\partial }{\partial \chi ^{i}}+{\mathcal {U}}_{i}\frac{\partial }{\partial {\mathcal {U}}}+{\mathcal {U}}_{ij}\frac{\partial }{\partial {\mathcal {U}}_{j}}+\cdots ,~~~i=1,2,3\dots m. \end{aligned}$$
2. Defining the Euler operator as below:
  38 $$\begin{aligned} \frac{{\delta }}{{\delta {\mathcal {U}}}}=\frac{\partial }{\partial {\mathcal {U}}}-D_{i}\frac{\partial }{\partial {\mathcal {U}}_{i}}+D_{ij}\frac{\partial }{\partial {\mathcal {U}}_{ij}}-D_{ijk}\frac{\partial }{\partial {\mathcal {U}}_{ijk}}+ \cdots ~. \end{aligned}$$
3. Let us define n-tuple ${\mathfrak {f}}=({\mathfrak {f}}^{1},{\mathfrak {f}}^{2},{\mathfrak {f}}^{3},\dots ,{\mathfrak {f}}^{m})$, $i=1,2,\dots m$,
  39 $$\begin{aligned} D_{i}{\mathfrak {f}}^{i}=0, \end{aligned}$$
  Equation (39) is said to be the conservation laws and it fulfils all results of Eq. (8).
4. The purpose of $\Lambda (\chi , \tau , {\mathcal {U}})$ of the Eq. (8):
  40 $$\begin{aligned} D_{i}{\mathfrak {f}}^{i}=\Lambda (\chi ,\tau ,{\mathcal {U}}) H, \end{aligned}$$
  for some function ${\mathcal {U}}(\mu ^{1},\mu ^{2}, \ldots ,\mu ^{m})$.
5. We will obtain the determining equations for $\Lambda (\chi , \tau , {\mathcal {U}})$ after calculating the derivative of $\Lambda (\chi , \tau , {\mathcal {U}})$ in Eq. (40):
  41 $$\begin{aligned} \frac{\delta }{\delta \mathcal {U}}(\Lambda (\chi , \tau , {\mathcal {U}}) H)=0. \end{aligned}$$
  Equation (41) depends for some function ${\mathcal {U}}(\mu ^{1}, \mu ^{2}, . . . , \mu ^{m})$. Finally, when we calculate the $\Lambda (X, t, {\mathcal {U}})$ with use of Eq. (41), the conservation laws can be acquired by Eq. (40).

## Lie group analysis of Eq. (6)

Here, we are supposing the Lie approach for assumed Eq. (6). Now, suppose the one-parameter Lie group of infinitesimal transformations on $(\tau ,\chi ,\mathcal {U})$ given by

$$\begin{aligned} \bar{\tau }=&\tau +\varepsilon ~\zeta ^{1}(\tau ,\chi ,\mathcal {U})+O(\varepsilon ^{2}),\\ \bar{\chi }=&\chi +\varepsilon ~\zeta ^{2}(\tau ,\chi ,\mathcal {U})+O(\varepsilon ^{2}),\\ \bar{\mathcal {U}}=&u+\varepsilon ~{\eta }(\tau ,\chi ,\mathcal {U})+O(\varepsilon ^{2}), \end{aligned}$$

and $\varepsilon \ll 1$ is a Small parameter. The associated Lie algebra of infinitesimal symmetries is generated by vector fields

42 $$\begin{aligned} \begin{aligned} \mathfrak {X}=&\zeta ^{1}(\tau ,\chi ,\mathcal {U})\partial _{\tau }+\zeta ^{2}(\tau ,\chi ,\mathcal {U})\partial _{\chi } +{\eta }(\tau ,\chi ,\mathcal {U})\partial _{\mathcal {U}}. \end{aligned} \end{aligned}$$

Equation (42) creates a symmetry of Eq. (6), and $\mathfrak {X}$ satisfy the Lie group conditions

$$\begin{aligned} Pr^{(4)}\mathfrak {X}\bigg (\frac{\partial ^2 {\mathcal {U}}}{\partial \tau ^2}=\delta ^2_{o}\frac{\partial ^2 {\mathcal {U}}}{\partial \chi ^2}+p_{o} \frac{\partial {\mathcal {U}}}{\partial \chi } \frac{\partial ^2 {\mathcal {U}}}{\partial \chi ^2}+q_{o} \bigg (\frac{\partial {\mathcal {U}}}{\partial \chi }\bigg )^2 \frac{\partial ^2 {\mathcal {U}}}{\partial \chi ^2}+r\frac{\partial ^4 {\mathcal {U}}}{\partial \chi ^4}\bigg )|_{{Eq.(6)}=0} =0. \end{aligned}$$

The fourth prolongation $Pr^{(4)}$ for $\mathfrak {X}$ can be written as:

43 $$\begin{aligned} \begin{aligned} Pr^{(4)}=&\mathfrak {X}+{\eta }^{\chi }\frac{\partial }{\partial {\mathcal {U}}_{\chi }}+{\eta }^{\tau \tau }\frac{\partial }{\partial {\mathcal {U}}_{\tau \tau }}+{\eta }^{\chi \chi }\frac{\partial }{\partial {\mathcal {U}}_{\chi \chi }}+ {\eta }^{\chi \chi \chi \chi }\frac{\partial }{\partial {\mathcal {U}}_{\chi \chi \chi \chi }}, \end{aligned} \end{aligned}$$

furthermore, we have

44 $$\begin{aligned} {\left\{ \begin{array}{ll} \eta ^{\chi }=D_{\chi }(\eta )-{{\mathcal {U}}}_{\chi }D_{\chi }(\zeta ^{1})-{{\mathcal {U}}}_{\tau }D_{\chi }(\zeta ^{2}),\\ \eta ^{\chi \chi }=D_{\chi }(\eta ^{\chi })-{{\mathcal {U}}}_{\chi \chi }D_{\chi }(\zeta ^{1})-{{\mathcal {U}}}_{\tau \chi }D_{\chi }(\zeta ^{2}),\\ \eta ^{\tau }=D_{\tau }(\eta )-{{\mathcal {U}}}_{\chi }D_{t}(\zeta ^{1})-{{\mathcal {U}}}_{\tau }D_{\tau }(\zeta ^{2}),\\ \eta ^{\tau \tau }=D_{\tau }(\eta ^{\tau })-{{\mathcal {U}}}_{\tau \tau }D_{\tau }(\zeta ^{1})-{{\mathcal {U}}}_{\chi \tau }D_{t}(\zeta ^{2}),\\ \eta ^{\chi \chi \chi }=D_{\chi }(\eta ^{\chi \chi })-{{\mathcal {U}}}_{\chi \chi \chi }D_{\chi }(\zeta ^{1})-{{\mathcal {U}}}_{\tau \chi \chi }D_{\chi }(\zeta ^{2}). \end{array}\right. } \end{aligned}$$

Let $(x^{1},x^{2})=(\chi ,\tau )$, where $D_{i}$ can be written as:

$$\begin{aligned} D_{i}=\frac{\partial }{\partial \chi ^{i}}+{{\mathcal {U}}}_{i}\frac{\partial }{\partial {{\mathcal {U}}}}+{{\mathcal {U}}}_{ij}\frac{\partial }{\partial {{\mathcal {U}}}_{j}}+\cdots ,~~~~ i=1,2. \end{aligned}$$

Substituting the values of ${\eta }^i$ which gives us the following vectors:

45 $$\begin{aligned} \mathfrak {X}_{1}=\frac{\partial }{\partial \chi },~~~~~~ \mathfrak {X}_{2}=\frac{\partial }{\partial \tau },~~~~~~\mathfrak {X}_{3}=\frac{\partial }{\partial \mathcal {U}},~~~~~~ \mathfrak {X}_{4}=\tau \frac{\partial }{\partial \mathcal {U}}. \end{aligned}$$

We see that

$$\begin{aligned}{}[\mathfrak {X}_{i},\mathfrak {X}_{j}]=0,~~~where~~i,j=1,2,3. \end{aligned}$$

## Optimal system

In this section, we observe that from the obtained vector field Eq. (45), the $\mathfrak {X}=\{\mathfrak {X}_1,\mathfrak {X}_2\}$ forms an abelian algebra. So we can use the (42) and get:

46 $$\begin{aligned} \begin{aligned} \pounds _{1}=&<\mathfrak {X}_1>,\\ \pounds _{2}=&<\mathfrak {X}_1+k_1 \mathfrak {X}_2>. \end{aligned} \end{aligned}$$

### Similarity reduction of Eq. (6)

Here, we will find the similarity variables and analytical results for Eq. (6).

#### $\pounds _{1}=< \mathfrak {X}_1>$

Using the vector $\pounds _{1}$, we get the new variable

47 $$\begin{aligned} {u(\tau ,\chi )}={\mathcal {H}}(\varrho ),~~~where~~\varrho =\chi , \end{aligned}$$

putting the (47) into Eq. (6), which gives us

48 $$\begin{aligned} \mathcal {U}(\tau ,\chi )= m_1 \tau +m_2, \end{aligned}$$

where $m_1$ and $m_2$ are integration constants.

#### $\pounds _{2}=<\mathfrak {X}_1+k_1 \mathfrak {X}_2$

Using the vector $\pounds _{2}$, we get the new variable

49 $$\begin{aligned} {u(\tau ,\chi )}={\mathcal {H}}(\varrho ),~~~where~~\varrho =\chi +k_1\tau , \end{aligned}$$

putting the (49) into Eq. (6), which gives us

50 $$\begin{aligned} 6 \left( k_1^2-\delta _o^2 \right) {\mathcal {H}}^{\prime }-3p_o \left( {\mathcal {H}}^{\prime } \right) ^2 -2q_o \left( {\mathcal {H}}^{\prime } \right) ^3-3r{\mathcal {H}}^{\prime \prime \prime }=0. \end{aligned}$$

### Application of new auxiliary method

Here, we aim to construct the wave patterns for Eq. (6) from Eq. (50) with the use of the proposed technique. Using the balancing method and we obtain $N=1$. Using the value of $N=1$ in (11) and we have

51 $$\begin{aligned} {\mathcal {H}}(\varrho )=C_o+C_1{\mathfrak {F}}^{q(\varrho )}. \end{aligned}$$

We have to put Eq. (51) into Eq. (50) and we get the system of the equation after comparing the coefficients of ${{\mathfrak {F}}^{q(\varrho )}}$. With the use of Maple, we solved the obtained system of equations and got the following results.

52 $$\begin{aligned} \begin{aligned} C_0=V_1, ~~~~C_1=-\frac{12r\mathfrak {B}_3}{p_o}, ~~~~k_1=\pm \sqrt{r\mathfrak {B}_1^2-4r\mathfrak {B}_2\mathfrak {B}_3+\delta _o^2}. \end{aligned} \end{aligned}$$

Using Eq. (52) into Eq. (51) gives us the following set of solutions.

53 $$\begin{aligned} \mathcal {U}(\chi ,\tau )=V_1-\frac{12r\mathfrak {B}_3}{p_o}{\mathfrak {F}}^{q(\varrho )},~~~~ \text {where}~~~~\varrho =\chi \pm \sqrt{r\mathfrak {B}_1^2-4r\mathfrak {B}_2\mathfrak {B}_3+\delta _o^2}~\tau . \end{aligned}$$

Where $V_1$ is an arbitrary constant.

- *Case 1* When ${\mathfrak {B}_{1}}^2-{\mathfrak {B}_{2}}{\mathcal {U}_{3}}<0$ and ${\mathfrak {B}_{3}}\ne 0$
  54 $$\begin{aligned} \mathcal {U}_1(\chi ,\tau )=V_1-\frac{12r\mathfrak {B}_3}{p_o} \left\{ \frac{-{\mathfrak {B}_{1}}}{{\mathfrak {B}_{3}}} +\frac{\sqrt{- \left( {\mathfrak {B}_{1}}^2-{\mathfrak {B}_{2}}{\mathfrak {B}_{3}} \right) }}{{\mathfrak {B}_{3}}}\tan \left( \frac{\sqrt{- \left( {\mathfrak {B}_{1}} ^2-{\mathfrak {B}_{2}}{\mathfrak {B}_{3}} \right) }}{2}\varrho \right) \right\} , \end{aligned}$$
  55 $$\begin{aligned} {\mathcal {U}}_2(\chi ,\tau )=V_1-\frac{12r\mathfrak {B}_3}{p_o}\left\{ \frac{-{\mathfrak {B}_{1}}}{{\mathfrak {B}_{3}}} +\frac{\sqrt{- \left( {\mathfrak {B}_{1}}^2-{\mathfrak {B}_{2}}{\mathfrak {B}_{3}} \right) }}{{\mathfrak {B}_{3}}}\cot \left( \frac{\sqrt{- \left( {\mathfrak {B}_{1}}^2-{\mathfrak {B}_{2}}{\mathfrak {B}_{3}} \right) }}{2}\varrho \right) \right\} . \end{aligned}$$
- *Case 2* When ${\mathfrak {B}_{1}}^2-{\mathfrak {B}_{2}}{\mathfrak {B}_{3}}>0$ and ${\mathfrak {B}_{3}}\ne 0$
  56 $$\begin{aligned} {\mathcal {U}}_3(\chi ,\tau )=V_1-\frac{12r\mathfrak {B}_3}{p_o} \left\{ \frac{-{\mathfrak {B}_{1}}}{{\mathfrak {B}_{3}}} +\frac{\sqrt{ \left( {\mathfrak {B}_{1}}^2-{\mathfrak {B}_{2}}{\mathfrak {B}_{3}} \right) }}{{\mathfrak {B}_{3}}}\tanh \left( \frac{\sqrt{ \left( {\mathfrak {B}_{1}} ^2-{\mathfrak {B}_{2}}{\mathfrak {B}_{3}} \right) }}{2}\varrho \right) \right\} , \end{aligned}$$
  57 $$\begin{aligned} {\mathcal {U}}_4(\chi ,\tau )=V_1-\frac{12r\mathfrak {B}_3}{p_o} \left\{ \frac{-{\mathfrak {B}_{1}}}{{\mathfrak {B}_{3}}} -\frac{\sqrt{ \left( {\mathfrak {B}_{1}}^2-{\mathfrak {B}_{2}}{\mathfrak {B}_{3}} \right) }}{{\mathfrak {B}_{3}}}\coth \left( \frac{\sqrt{ \left( {\mathfrak {B}_{1}} ^2-{\mathfrak {B}_{2}}{\mathfrak {B}_{3}} \right) }}{2}\varrho \right) \right\} . \end{aligned}$$
- *Case 3* When ${\mathfrak {B}_{1}}^2+{\mathfrak {B}_{2}}{\mathfrak {B}_{3}}>0$ and ${\mathfrak {B}_{3}}\ne 0$ and ${\mathfrak {B}_{3}}\ne -{\mathfrak {B}_{2}}$
  58 $$\begin{aligned} {\mathcal {U}}_5(\chi ,\tau )=V_1-\frac{12r\mathfrak {B}_3}{p_o} \left\{ \frac{{\mathfrak {B}_{1}}}{{\mathfrak {B}_{3}}} +\frac{\sqrt{ \left( {\mathfrak {B}_{1}}^2 +{\mathfrak {B}_{2}}^2 \right) }}{{\mathfrak {B}_{3}}}\tanh \left( \frac{\sqrt{({\mathfrak {B}_{1}}^2 +{\mathfrak {B}_{2}}^2)}}{2}\varrho \right) \right\} , \end{aligned}$$
  59 $$\begin{aligned} {\mathcal {U}}_6(\chi ,\tau )=V_1-\frac{12r\mathfrak {B}_3}{p_o} \left\{ \frac{{\mathfrak {B}_{1}}}{{\mathfrak {B}_{3}}} +\frac{\sqrt{ \left( {\mathfrak {B}_{1}}^2+{\mathfrak {B}_{2}}^2 \right) }}{{\mathfrak {B}_{3}}}\coth \left( \frac{\sqrt{ \left( {\mathfrak {B}_{1}}^2+{\mathfrak {B}_{2}}^2 \right) }}{2}\varrho \right) \right\} . \end{aligned}$$
- *Case 4* When ${\mathfrak {B}_{1}}^2+{\mathfrak {B}_{2}}{\mathfrak {B}_{3}}<0$, ${\mathfrak {B}_{3}}\ne 0$ and ${\mathfrak {B}_{3}}\ne -{\mathfrak {B}_{2}}$
  60 $$\begin{aligned} {\mathcal {U}}_7(\chi ,\tau )=V_1-\frac{12r\mathfrak {B}_3}{p_o} \left\{ \frac{{\mathfrak {B}_{1}}}{{\mathfrak {B}_{3}}}+\frac{\sqrt{- \left( {\mathfrak {B}_{1}}^2 +{\mathfrak {B}_{2}}^2 \right) }}{{\mathfrak {B}_{3}}}\tan \left( \frac{\sqrt{- \left( {\mathfrak {B}_{1}}^2+{\mathfrak {B}_{2}}^2 \right) }}{2}\varrho \right) \right\} , \end{aligned}$$
  61 $$\begin{aligned} {\mathcal {U}}_8(\chi ,\tau )=V_1-\frac{12r\mathfrak {B}_3}{p_o} \left\{ \frac{{\mathfrak {B}_{1}}}{{\mathfrak {B}_{3}}} +\frac{\sqrt{- \left( {\mathfrak {B}_{1}}^2+{\mathfrak {B}_{2}}^2 \right) }}{{\mathfrak {B}_{3}}}\cot \left( \frac{\sqrt{- \left( {\mathfrak {B}_{1}}^2+{\mathfrak {B}_{2}}^2 \right) }}{2}\varrho \right) \right\} . \end{aligned}$$
- *Case 5* When ${\mathfrak {B}_{1}}^2-{\mathfrak {B}_{2}}^2<0$ and ${\mathfrak {B}_{3}}\ne -{\mathfrak {B}_{2}}$
  62 $$\begin{aligned} {\mathcal {U}}_9(\chi ,\tau )=V_1-\frac{12r\mathfrak {B}_3}{p_o} \left\{ \frac{-{\mathfrak {B}_{1}}}{{\mathfrak {B}_{3}}}+\frac{\sqrt{- \left( {\mathfrak {B}_{1}}^2 -{\mathfrak {B}_{2}}^2 \right) }}{{\mathfrak {B}_{3}}}\tan \left( \frac{\sqrt{- \left( {\mathfrak {B}_{1}}^2-{\mathfrak {B}_{2}}^2 \right) }}{2}\varrho \right) \right\} , \end{aligned}$$
  63 $$\begin{aligned} {\mathcal {U}}_{10}(\chi ,\tau )=V_1-\frac{12r\mathfrak {B}_3}{p_o} \left\{ \frac{-{\mathfrak {B}_{1}}}{{\mathfrak {B}_{3}}} +\frac{\sqrt{- \left( {\mathfrak {B}_{1}}^2-{\mathfrak {B}_{2}}^2 \right) }}{{\mathfrak {B}_{3}}}\cot \left( \frac{\sqrt{- \left( {\mathfrak {B}_{1}}^2-{\mathfrak {B}_{2}}^2 \right) }}{2}\varrho \right) \right\} . \end{aligned}$$
- *Case 6* When ${\mathfrak {B}_{1}}^2-{\mathfrak {B}_{2}}^2>0$ and ${\mathfrak {B}_{3}}\ne -{\mathfrak {B}_{2}}$
  64 $$\begin{aligned} {\mathcal {U}}_{11}(\chi ,\tau )=V_1-\frac{12r\mathfrak {B}_3}{p_o} \left\{ \frac{-{\mathfrak {B}_{1}}}{{\mathfrak {B}_{3}}} +\frac{\sqrt{ \left( {\mathfrak {B}_{1}}^2-{\mathfrak {B}_{2}}^2 \right) }}{{\mathfrak {B}_{3}}}\tanh \left( \frac{\sqrt{ \left( {\mathfrak {B}_{1}}^2-{\mathfrak {B}_{2}}^2 \right) }}{2}\varrho \right) \right\} , \end{aligned}$$
  65 $$\begin{aligned} {\mathcal {U}}_{12}(\chi ,\tau )=V_1-\frac{12r\mathfrak {B}_3}{p_o} \bigg \{\frac{-{\mathfrak {B}_{1}}}{{\mathfrak {B}_{3}}} +\frac{\sqrt{({\mathfrak {B}_{1}}^2-{\mathfrak {B}_{2}}^2)}}{{\mathfrak {B}_{3}}}\coth \bigg (\frac{\sqrt{({\mathfrak {B}_{1}}^2-{\mathfrak {B}_{2}}^2)}}{2}\varrho \bigg )\bigg \}. \end{aligned}$$
- *Case 7* When ${\mathfrak {B}_{2}}{\mathfrak {B}_{3}}>0$, ${\mathfrak {B}_{3}}\ne 0$ and ${\mathfrak {B}_{1}}=0$
  66 $$\begin{aligned} {\mathcal {U}}_{13}(\chi ,\tau )=V_1-\frac{12r\mathfrak {B}_3}{p_o} \left\{ \sqrt{\frac{-{\mathfrak {B}_{2}}}{{\mathfrak {B}_{3}}}}\tanh \bigg (\frac{\sqrt{ -{\mathfrak {B}_{2}}{\mathfrak {B}_{3}}}}{2}\varrho \bigg ) \right\} , \end{aligned}$$
  67 $$\begin{aligned} {\mathcal {U}}_{14}(\chi ,\tau )=V_1-\frac{12r\mathfrak {B}_3}{p_o} \left\{ \sqrt{\frac{-{\mathfrak {B}_{2}}}{{\mathfrak {B}_{3}}}}\coth \bigg (\frac{\sqrt{-{\mathfrak {B}_{2}}{\mathfrak {B}_{3}}}}{2}\varrho \bigg ) \right\} . \end{aligned}$$
- *Case 8* When ${\mathfrak {B}_{1}}=0$ and ${\mathfrak {B}_{2}}=-{\mathfrak {B}_{3}}$
  68 $$\begin{aligned} {\mathcal {U}}_{15}(\chi ,\tau )=V_1-\frac{12r\mathfrak {B}_3}{p_o} \bigg \{\frac{-(1+e^{2{\mathfrak {B}_{2}}\varrho })\pm \sqrt{2(1+e^{2{\mathfrak {B}_{2}}\varrho })}}{e^{2{\mathfrak {B}_{2}}\varrho }-1}\bigg \}. \end{aligned}$$
- *Case 9* When $${\mathfrak {B}_{1}}^2={\mathfrak {B}_{2}}{\mathfrak {B}_{3}}$$
  69 $$\begin{aligned} {\mathcal {U}}_{16}(\chi ,\tau )=V_1-\frac{12r\mathfrak {B}_3}{p_o} \bigg \{\frac{-{\mathfrak {B}_{2}}({\mathfrak {B}_{1}}\varrho +2)}{{\mathfrak {B}_{1}}^2\varrho }\bigg \}. \end{aligned}$$
- *Case 10* When ${\mathfrak {B}_{1}}=k$, ${\mathfrak {B}_{2}}=2k$ and ${\mathfrak {B}_{3}}=0$
  70 $$\begin{aligned} {\mathcal {U}}_{17}(\chi ,\tau )=V_1-\frac{12r\mathfrak {B}_3}{p_o}\bigg \{e^{\varrho }-1\bigg \}. \end{aligned}$$
- *Case 11* When ${\mathfrak {B}_{1}}=k$, ${\mathfrak {B}_{3}}=2k$ and ${\mathfrak {B}_{2}}=0$
  71 $$\begin{aligned} {\mathcal {U}}_{18}(\chi ,\tau )=V_1-\frac{12r\mathfrak {B}_3}{p_o}\bigg \{\frac{e^{\varrho }}{1-e^{\varrho }}\bigg \}. \end{aligned}$$
- *Case 12* When $$2{\mathfrak {B}_{1}}={\mathfrak {B}_{2}}+{\mathfrak {B}_{3}}$$
  72 $$\begin{aligned} {\mathcal {U}}_{19}(\chi ,\tau )=V_1-\frac{12r\mathfrak {B}_3}{p_o}\bigg \{\frac{1+{\mathfrak {B}_{2}} e^{\frac{1}{2}({\mathfrak {B}_{2}}-{\mathfrak {B}_{3}})\varrho }}{ 1+{\mathfrak {B}_{3}} e^{\frac{1}{2}({\mathfrak {B}_{2}}-{\mathfrak {B}_{3}})\varrho }}\bigg \}. \end{aligned}$$
- *Case 13* When $$-2{\mathfrak {B}_{1}}={\mathfrak {B}_{2}}+{\mathfrak {B}_{3}}$$
  73 $$\begin{aligned} {\mathcal {U}}_{20}(\chi ,\tau )=V_1-\frac{12r\mathfrak {B}_3}{p_o}\bigg \{\frac{{\mathfrak {B}_{2}}+{\mathfrak {B}_{2}} e^{\frac{1}{2}({\mathfrak {B}_{2}}-{\mathfrak {B}_{3}})\varrho }}{ {\mathfrak {B}_{3}}+{\mathfrak {B}_{3}} e^{\frac{1}{2}({\mathfrak {B}_{2}}-{\mathfrak {B}_{3}})\varrho }}\bigg \}. \end{aligned}$$
- *Case 14* When $${\mathfrak {B}_{2}}=0$$
  74 $$\begin{aligned} {\mathcal {U}}_{21}(\chi ,\tau )=V_1-\frac{12r\mathfrak {B}_3}{p_o}\bigg \{\frac{{\mathfrak {B}_{1}} e^{{\mathfrak {B}_{1}}\varrho }}{ 1+\frac{{\mathfrak {B}_{3}}}{2}e^{{\mathfrak {B}_{1}}\varrho }}\bigg \}. \end{aligned}$$
- *Case 15* When $${\mathfrak {B}_{2}}={\mathfrak {B}_{1}}={\mathfrak {B}_{3}}\ne 0$$
  75 $$\begin{aligned} {\mathcal {U}}_{22}(\chi ,\tau )=V_1-\frac{12r\mathfrak {B}_3}{p_o} \bigg \{\frac{-({\mathfrak {B}_{2}}\varrho +2)}{{\mathfrak {B}_{2}}\varrho }\bigg \}. \end{aligned}$$
- *Case 16* When $${\mathfrak {B}_{2}}={\mathfrak {B}_{3}}$$, $${\mathfrak {B}_{1}}=0$$
  76 $$\begin{aligned} {\mathcal {U}}_{23}(\chi ,\tau )=V_1-\frac{12r\mathfrak {B}_3}{p_o} \bigg \{\tan \bigg (\frac{{\mathfrak {B}_{2}}\varrho +c}{2}\bigg )\bigg \}. \end{aligned}$$
- *Case 17* When $${\mathfrak {B}_{3}}=0$$
  77 $$\begin{aligned} {\mathcal {U}}_{24}(\chi ,\tau )=V_1-\frac{12r\mathfrak {B}_3}{p_o} \bigg \{e^{{\mathfrak {B}_{1}}\varrho }-\frac{{\mathfrak {B}_{2}}}{2{\mathfrak {B}_{1}}}\bigg \}. \end{aligned}$$
  where $\varrho =\chi \pm \sqrt{r\mathfrak {B}_1^2-4r\mathfrak {B}_2\mathfrak {B}_3+\delta _o^2}~\tau$ is given according to.

**Figure 1:**
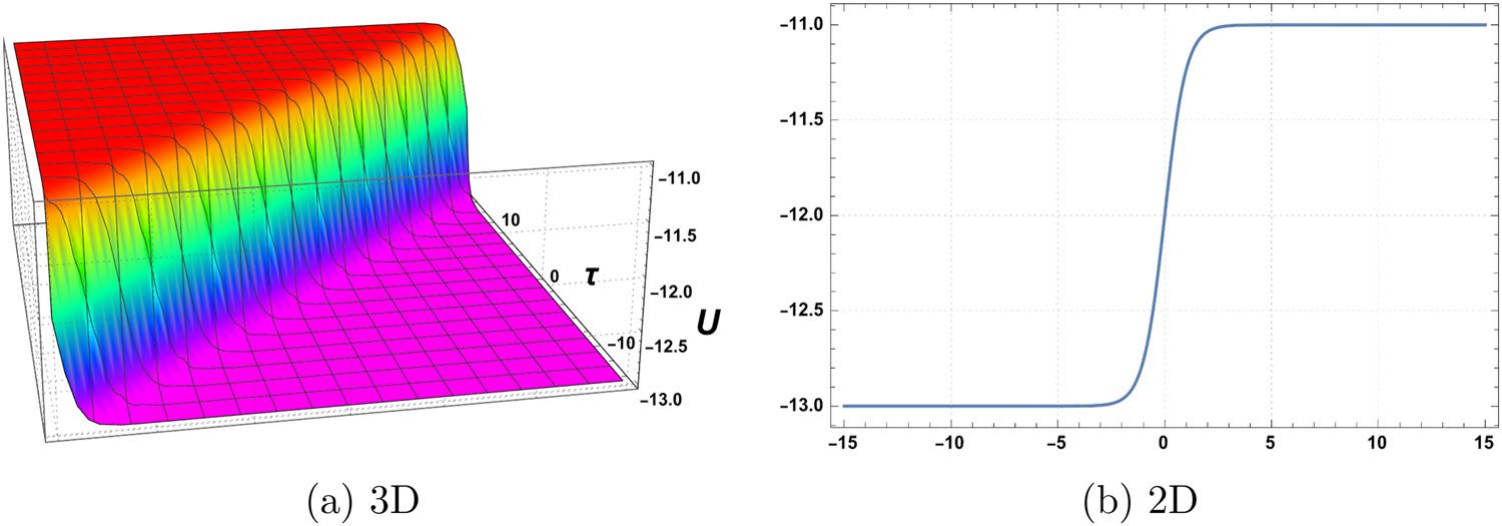
Graphics of (**a**) $\mathcal {U}_{1}(\chi , \tau )$ versus time component $\tau$ for the choice of parameters $\delta _o=1$, $r=2$, $p_o=3$, $\mathfrak {B}_{1}=2$, $\mathfrak {B}_{2}=1$,$\mathfrak {B}_{3}=2$ and (**b**) considering at $\tau =1$.

**Figure 2:**
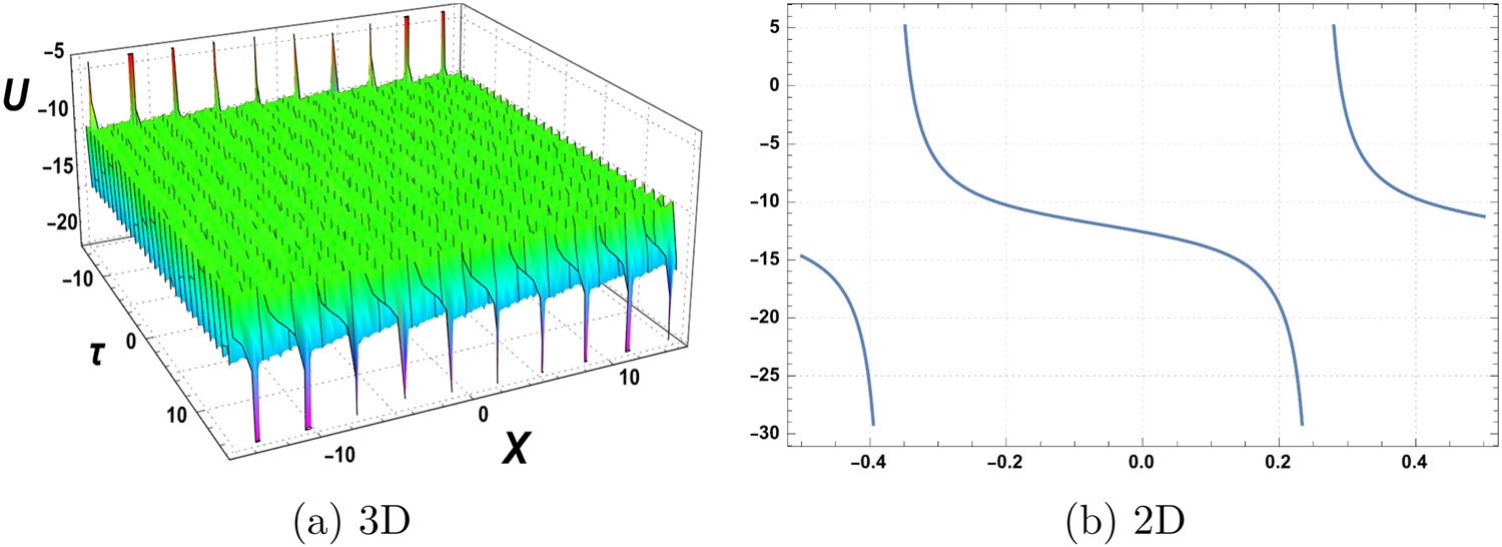
Graphics of (**a**) $\mathcal {U}_{2}(\chi , \tau )$ versus time component $\tau$ for the choice of parameters $\delta _o=2$, $r=1$, $p_o=1$, $\mathfrak {B}_{1}=3$, $\mathfrak {B}_{2}=2$,$\mathfrak {B}_{3}=1$, and (**b**) considering at $\tau =2$.

**Figure 3:**
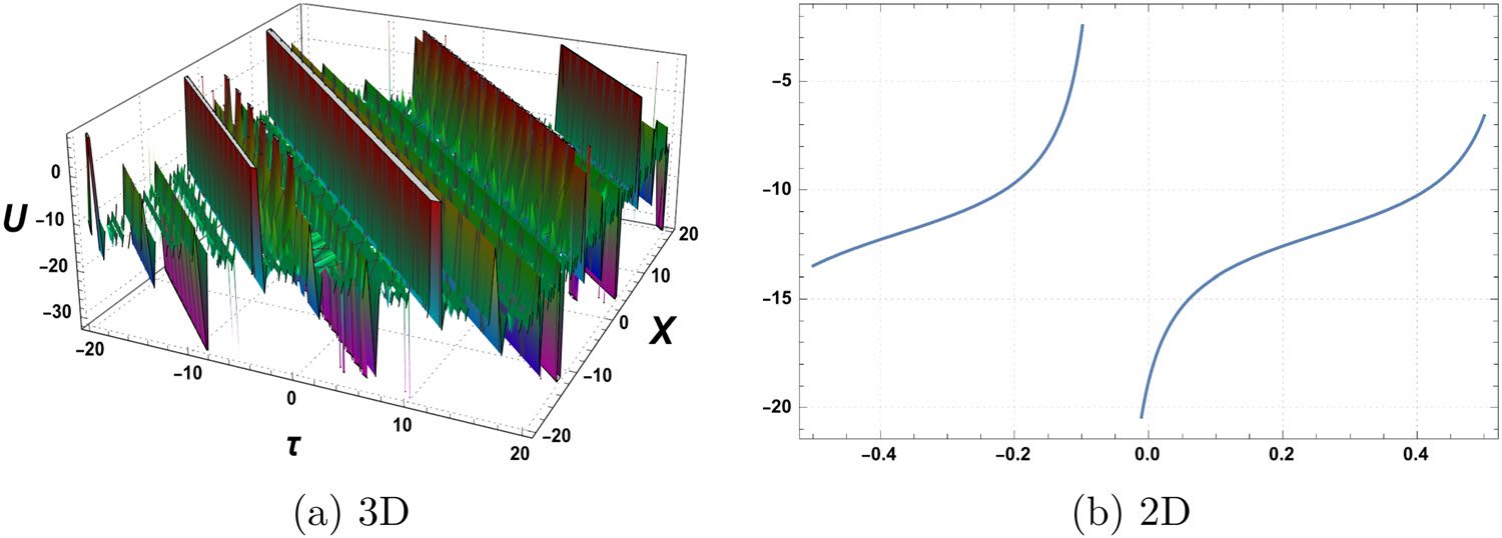
Graphics of (**a**) $\mathcal {U}_{3}(\chi , \tau )$ versus time component $\tau$ for the choice of parameters $\delta _o=1.5$, $r=1$, $p_o=4$, $\mathfrak {B}_{1}=4$, $\mathfrak {B}_{2}=3$,$\mathfrak {B}_{3}=5$, and (**b**) considering at $\tau =2$.

**Figure 4:**
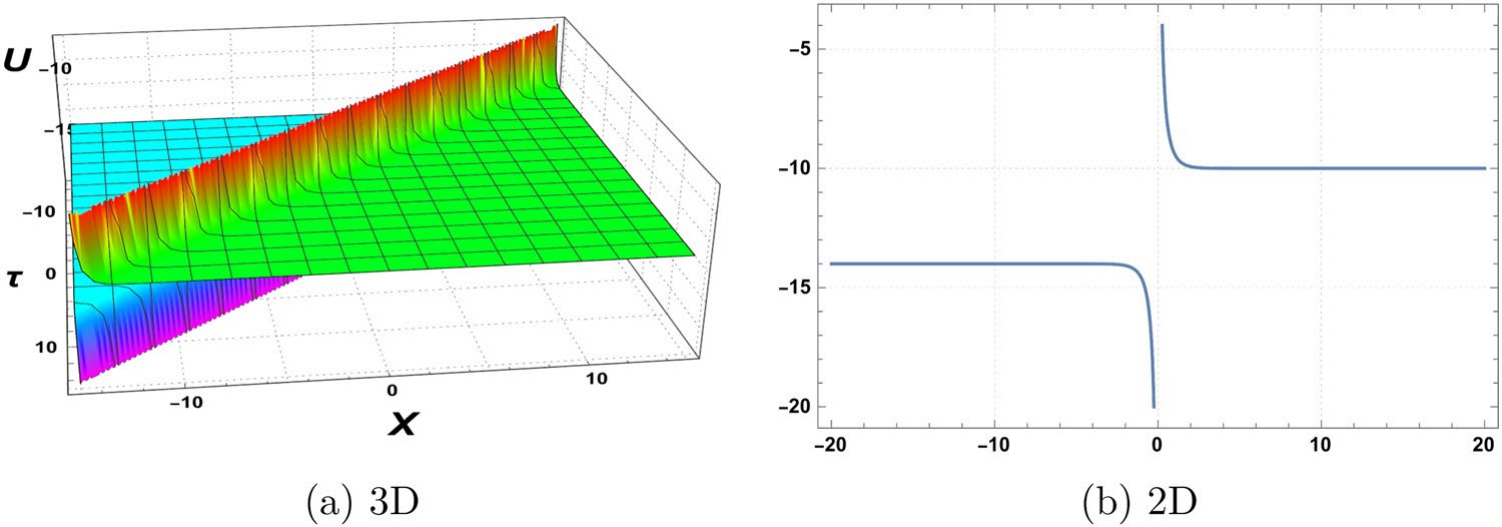
Graphics of (**a**) $\mathcal {U}_{4}(\chi , \tau )$ versus time component $\tau$ for the choice of parameters $\delta _o=5$, $r=0.5$, $p_o=3.5$, $\mathfrak {B}_{1}=1.5$, $\mathfrak {B}_{2}=3.5$,$\mathfrak {B}_{3}=2.5$, and (**b**) considering at $\tau =3$.

**Figure 5:**
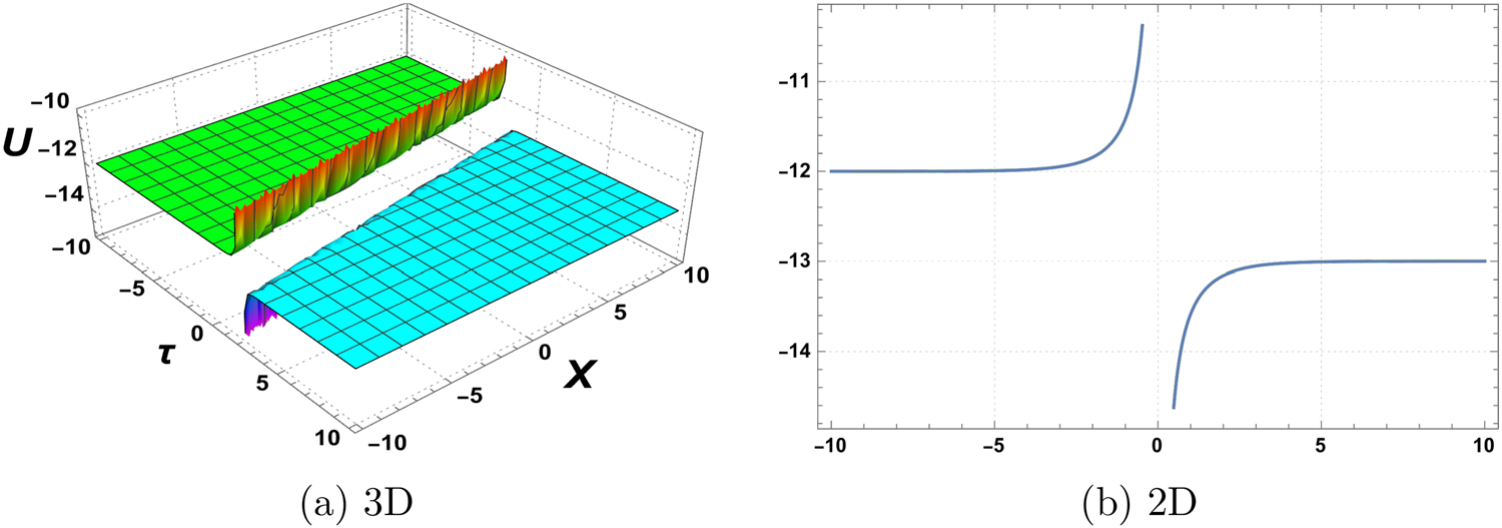
Graphics of (**a**) $\mathcal {U}_{19}(\chi , \tau )$ versus time component $\tau$ for the choice of parameters $\delta _o=6$, $r=3$, $p_o=5$, $\mathfrak {B}_{1}=1$, $\mathfrak {B}_{2}=3$,$\mathfrak {B}_{3}=5$, and (**b**) considering at $\tau =5$.

## Graphics and discussion

A graphical representation of obtained solutions is discussed here in this section. By using the new auxiliary method we have constructed the analytical behaviour of the considered model in the form of trigonometric functions, hyperbolic trigonometric functions, and exponential, and algebraic-type results. The graph of the tangent function is periodic with a period of $\Pi$ and has vertical asymptotes at odd multiples of $\frac{\Pi }{2}$. As $\chi$ approaches these vertical asymptotes, the tangent function approaches positive or negative infinity depending on the direction of the approach. The graph of the cotangent function is also periodic with a period of $\Pi$ and has horizontal asymptotes at even multiples of $\Pi$. As $\chi$ approaches these horizontal asymptotes, the cotangent function approaches zero. We have plotted the behaviour of some obtained results. Figure 1 shows the graphical behavior of $u_{1}(\chi , \tau )$ for the choice of parameters $\delta _o=1$, $r=2$, $p_o=3$, $\mathfrak {B}_{1}=2$, $\mathfrak {B}_{2}=1$,$\mathfrak {B}_{3}=2$, $\tau =1$. Figure 2 represent the behaviour of $u_{2}(\chi , \tau )$ for the choice of parameters $\delta _o=2$, $r=1$, $p_o=1$, $\mathfrak {B}_{1}=3$, $\mathfrak {B}_{2}=2$,$\mathfrak {B}_{3}=1$, $\tau =2$. Figure 3 shows the Graphics of $u_{3}(\chi , \tau )$ for the choice of parameters $\delta _o=1.5$, $r=1$, $p_o=4$, $\mathfrak {B}_{1}=4$, $\mathfrak {B}_{2}=3$,$\mathfrak {B}_{3}=5$, $\tau =2$. Figure 4 shows the Graphics of $u_{4}(\chi , \tau )$ for the choice of parameters $\delta _o=5$, $r=0.5$, $p_o=3.5$, $\mathfrak {B}_{1}=1.5$, $\mathfrak {B}_{2}=3.5$,$\mathfrak {B}_{3}=2.5$, $\tau =3$. Figure 5 represent the behaviour of $u_{19}(\chi , \tau )$ for the choice of parameters $\delta _o=6$, $r=3$, $p_o=5$, $\mathfrak {B}_{1}=1$, $\mathfrak {B}_{2}=3$,$\mathfrak {B}_{3}=5$, $\tau =5$. The behaviour of $\mathcal {U}_{19}$ shows Singularities exist in exponential functions due to their intrinsic nature of rapid and unbounded growth or decay. The exponential function, typically represented as $f(\chi ) = e^\chi$,where e is Euler’s number approximately equal to 2.71828,captures the exponential growth or decay phenomenon. However, certain values of $\chi$ result in problematic behaviour or undefined outcomes. When $\chi$ approaches positive or negative infinity, the exponential function exhibits an asymptotic behaviour, approaching infinity or zero, respectively, without reaching a definite value. These instances represent singularities where the exponential function becomes indeterminate or encounters difficulties in providing a well-defined result. These singularities in the exponential function highlight the inherent limitations and special characteristics associated with exponential growth or decay processes.

## Conservation laws

In this portion, we will construct the conservation laws by multiplier approach for Eq. (6). We obtain the determinant equation for $\Lambda (X, t, u)$ by Eq. (41).

78 $$\begin{aligned} \frac{\delta }{\delta \mathcal {U}}\bigg [\Lambda \bigg (\frac{\partial ^2 {\mathcal {U}}}{\partial \tau ^2}-\delta ^2_{o}\frac{\partial ^2 {\mathcal {U}}}{\partial \chi ^2}-p_{o} \frac{\partial {\mathcal {U}}}{\partial \chi } \frac{\partial ^2 {\mathcal {U}}}{\partial \chi ^2}-q_{o} \bigg (\frac{\partial {\mathcal {U}}}{\partial \chi }\bigg )^2 \frac{\partial ^2 {\mathcal {U}}}{\partial \chi ^2}-r\frac{\partial ^4 {\mathcal {U}}}{\partial \chi ^4}\bigg )\bigg ]=0. \end{aligned}$$

Using Eq. (38), we can write the Euler operator is of the form

79 $$\begin{aligned} \begin{aligned} \frac{\delta }{\delta \mathcal {U}}&=\frac{\partial }{\partial \mathcal {U}}-D_{\tau }\frac{\partial }{\partial \mathcal {U}_{\tau }}-D_{\chi }\frac{\partial }{\partial \mathcal {U}_{\chi }}+D^{2}_{\tau }\frac{\partial }{\partial \mathcal {U}_{\tau \tau }}+D^{2}_{\chi }\frac{\partial }{\partial \mathcal {U}_{\chi \chi }}+D_{\chi }D_{\tau }\frac{\partial }{\partial \mathcal {U}_{\tau \chi }} - \cdots , \end{aligned} \end{aligned}$$

defining the total derivative operators $D_{\tau }$ and $D_{\chi }$ from Eq. (37).

80 $$\begin{aligned} \begin{aligned} D_{\tau }&=\frac{\partial }{\partial \tau }+\mathcal {U}_{\tau }\frac{\partial }{\partial \mathcal {U}}+\mathcal {U}_{\tau \tau }\frac{\partial }{\partial \mathcal {U}_{\tau }}+\mathcal {U}_{\tau \chi }\frac{\partial }{\partial \mathcal {U}_{\chi }}\cdots ,\\ D_{\chi }&=\frac{\partial }{\partial \chi }+\mathcal {U}_{\chi }\frac{\partial }{\partial \mathcal {U}}+\mathcal {U}_{\chi \chi }\frac{\partial }{\partial \mathcal {U}_{\chi }}+\mathcal {U}_{\tau \chi }\frac{\partial }{\partial \mathcal {U}_{\tau }}\cdots , \end{aligned} \end{aligned}$$

computing Eq. (78) and we obtain the following multipliers and conservation laws:

81 $$\begin{aligned} \Lambda =C_1 \tau +C_2, \end{aligned}$$

by Eqs. (40) and (81), The following conservation laws are found

82 $$\begin{aligned} \begin{aligned} \mathcal {T}^\tau&=\mathcal {U}_\tau (C_1\tau +C_2)-C_1\mathcal {U},\\ \mathcal {T}^\chi&=(C_1\tau +C_2)\bigg (\frac{-1}{3}\mathcal {U}_\chi ^3q_o -\frac{1}{2}\mathcal {U}_\chi ^2p_o-\mathcal {U}_\chi \delta ^2_o-r\mathcal {U}_{\chi \chi \chi }\bigg ). \end{aligned} \end{aligned}$$

Using Eq. (82) and we get the following two cases of conservation laws.

- *Case 1* For $C_1=1,~C_2=0$, then $\Lambda _{1}=\tau$, we get the following fluxes:
  83 $$\begin{aligned} \begin{aligned} \mathcal {T}^\tau _1&=\tau \mathcal {U}_\tau -\mathcal {U},\\ \mathcal {T}^\chi _1&=\frac{-1}{3}\mathcal {U}_\chi ^3\tau q_o-\frac{1}{2}\mathcal {U}_\chi ^2\tau p_o-\mathcal {U}_\chi \tau \delta ^2_o-r\mathcal {U}_{\chi \chi \chi }\tau . \end{aligned} \end{aligned}$$
- *Case 2* For $C_1=0,~C_2=1$, then $\Lambda _{1}=1$, we get the following fluxes:
  84 $$\begin{aligned} \begin{aligned} \mathcal {T}^\tau _2&=\mathcal {U}_\tau ,\\ \mathcal {T}^\chi _2&=\frac{-1}{3}\mathcal {U}_\chi ^3 q_o-\frac{1}{2}\mathcal {U}_\chi ^2 p_o-\mathcal {U}_\chi \delta ^2_o-r\mathcal {U}_{\chi \chi \chi }. \end{aligned} \end{aligned}$$

## Conclusion

In this research, nonlinear chains of atoms(NCA) are studied. NCA play a crucial role in materials design and fabrication, enabling the development of advanced materials with tailored properties. The unique structural arrangements and interactions of nonlinear chains of atoms influence the mechanical, electrical, and optical properties of materials, which are essential for designing innovative technologies in fields such as nanotechnology, optoelectronics, and energy systems. Also, NCA are of great interest in the study of dynamical systems and nonlinear phenomena. They provide valuable insights into the behaviour of complex systems, allowing for the analysis and modelling of intricate dynamics observed in areas like physics, biology, and control theory. The Lie symmetry method and NAM enhances existing techniques, offering additional insights, improved accuracy, or simplified computations. Travelling wave solutions describe wave-like behaviour propagating through systems, while graphical behaviour provides visual representations of relationships and patterns in data or mathematical models. The multiplier method allows for the identification of conservation laws, which are fundamental principles in physics that state certain quantities remain constant over time. Understanding conservation laws and utilizing mathematical techniques such as the Lie symmetry method, travelling wave solutions, and graphical analysis contributes to a deeper understanding of nonlinear chains of atoms and their dynamics.

## Data Availability

All data that support the findings of this study are included within the article.
